# Review: Gut Microbiota—A Powerful Tool for Improving Pig Welfare by Influencing Behavior Through the Gut–Brain Axis

**DOI:** 10.3390/ani15131886

**Published:** 2025-06-26

**Authors:** Xiaoying Jian, Duo Zheng, Shengping Pang, Peiqiang Mu, Jun Jiang, Xu Wang, Xiliang Yan, Yinbao Wu, Yan Wang

**Affiliations:** 1 State Key Laboratory of Swine and Poultry Breeding Industry, College of Animal Science, South China Agricultural University, Guangzhou 510642, China; xiaoyingjian1999@163.com (X.J.); 13536760824@163.com (D.Z.); 18953636171@163.com (S.P.); yanxiliang1991@163.com (X.Y.); wuyinbao@scau.edu.cn (Y.W.); 2 College of Life Science, South China Agricultural University, Guangzhou 510642, China; mpeiqiang@scau.edu.cn (P.M.); jiangjun@scau.edu.cn (J.J.); 3 Institute of Quality Standard and Monitoring Technology for Agro-Products of Guangdong Academy of Agricultural Sciences, Guangzhou 510642, China; wangxuguangzhou@126.com; 4 Guangdong Provincial Key Lab of Agro-Animal Genomics and Molecular Breeding, South China Agricultural University, Guangzhou 510642, China; 5 National Engineering Research Center for Breeding Swine Industry, South China Agricultural University, Guangzhou 510642, China

**Keywords:** pig, behavior, microbiota–gut–brain axis, welfare

## Abstract

Animal welfare is one of the core concerns in the field of animal science, with behavior serving as a direct reflection of emotional state and health, and thus a key indicator for welfare assessment. With the widespread adoption of intensive farming systems, abnormal behaviors in pigs have become a prominent marker of compromised welfare. In the past few years, the role of gut microbes in the regulation of animal behavior has received increasing attention. This review summarizes the strong relationship between pig behavior and welfare, and focuses on the emerging research linking gut microbiota to behavioral expression in pigs. Furthermore, it outlines the mechanisms by which the microbiota modulates behavior through the microbiota–gut–brain axis (MGBA), including immune, endocrine, and neural pathways. Additionally, the potential of microbiota-targeted interventions to improve pig welfare, including probiotics and prebiotics, will be evaluated. As a critical bridge connecting physiology and psychology, the gut microbiota shows significant promise for advancing welfare regulation in pigs.

## Simple Summary

With the widespread adoption of intensive farming systems, pigs frequently exhibit various abnormal behaviors, which are widely regarded as indicators of compromised animal welfare. As behavior is a direct reflection of an animal’s emotional and physiological state, the presence of such behaviors implies impaired welfare. Increasing evidence suggests that pig behavior is influenced by the gut microbiota, primarily through the microbiota–gut–brain axis, involving immune, endocrine, and neural pathways. Therefore, modulating gut microbiota represents a promising strategy to improve pig welfare, including direct and indirect interventions.

## 1. Introduction

**Figure 1:**
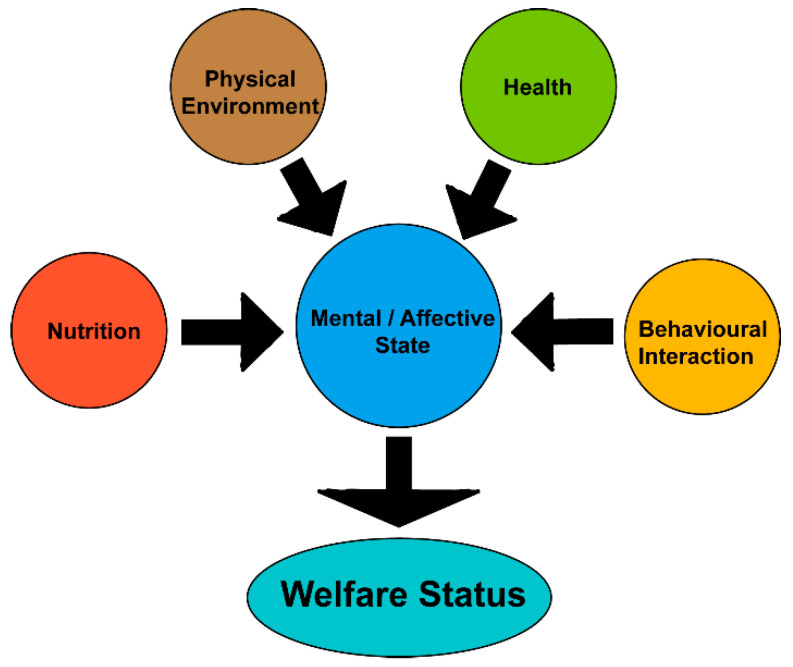
Linkages between the five domains of animal welfare.

With the growing global population, the demand for pork continues to rise, driving the swine industry toward more intensive farming systems. While such systems improve spatial efficiency, they are also associated with a range of behavioral problems in pigs, including tail biting (TB), bar biting, and abnormal postural behaviors such as excessive lying or standing. Multiple factors, such as disease, emotional state, and living conditions, can influence behavior [1]. Therefore, behavior can reflect physiological health, which, coupled with its ease of observation, is considered as one of the most effective indicators for assessing animal welfare. The presence of abnormal behaviors is generally regarded as a manifestation of compromised welfare in pigs. Animal welfare has significant implications for health status and product quality, thereby affecting production efficiency, which has attracted increasing attention. Furthermore, as education and economic development have progressed, public concern for animal welfare has grown substantially. The Farm Animal Welfare Council has proposed the “Five Freedoms” as a framework for animal welfare: freedom from hunger and thirst, discomfort, pain, injury and disease, fear and distress, and freedom to express normal behavior (Figure 1). An animal’s affective state is influenced by nutrition, physical environment, health, and behavioral interaction, and their interaction determines welfare status. This concept emphasizes not only physical health but also the psychological well-being of animals. Changes in psychological state are often difficult to assess through conventional physiological indicators but can be indirectly evaluated through alterations in behavior. With advances in technology and the emergence of smart farming systems, behavior monitoring methods have become increasingly sophisticated. These developments enable early detection and intervention of abnormal behaviors in pigs, which contributes to improved animal welfare and supports the sustainable development and productivity of the pig industry.

Behavior is regulated by the nervous system, which relies on neurotransmitters, including serotonin (5-hydroxytryptamine, 5-HT) and catecholamines. In response to stimuli, the levels of neurotransmitters such as γ-aminobutyric acid (GABA), 5-HT, and norepinephrine in the spinal cord undergo significant changes [2]. Enteroendocrine cells in the gut are capable of forming synaptic connections with vagal neurons, releasing neurotransmitters such as tryptophan to transmit sensory information [3,4]. This interaction establishes the gut–brain axis, a signaling pathway that links the gastrointestinal system with the brain, which impacts brain functions and behaviors. Conversely, neurotransmitters can also modulate gastrointestinal activity, influencing the gut immune system as well as microbiota composition [5]. Thus, neurotransmitters serve as key regulators in maintaining the bidirectional communication between gut and brain.

Gut microbiota, as a crucial component of the gut ecosystem, can also influence host behavior, particularly in areas such as depression, cognition, and social interaction [6,7,8]. One mechanism involves microbial metabolites that directly affect the central nervous system (CNS). The blood–brain barrier (BBB) can restrict the entry and exit of substances, whereas short-chain fatty acids (SCFAs) can pass through and alleviate depressive symptoms [9]. *Bacteroides ovatus* and its metabolite lysophosphatidylcholine have been shown to significantly reduce β-amyloid accumulation and improve cognitive impairment [8]. In addition, gut microbiota are capable of producing neurotransmitters. Genetically engineered Escherichia coli Nissle 1917, which synthesizes 5-HT, orally administered to constipated mice alleviated both constipation and depression-like behaviors [10]. Some microbial strains can convert glutamate into GABA [11]. *Lactobacillus brevis* DPC6108 and DSM32386 can produce GABA, thereby ameliorating metabolic syndrome, improving depressive behaviors, and lowering cortisol levels [12]. *Bacteroides*, *Parabacteroides*, and *Escherichia* also produce GABA, and the relative abundance of *Bacteroides* is negatively correlated with the brain characteristics associated with depression [13]. Furthermore, gut microbiota can influence synaptic activity and microglial activation within the nervous system, thereby modulating behaviors such as risk-taking and exploratory activity [1,14,15]. These findings have led to an extension of the gut–brain axis concept into the “microbiota–gut–brain” (MGBA).

In the midst of tremendous changes in agriculture, it is crucial not only to accelerate intensive farming, but also continue to evaluate, review, and enhance the welfare of pigs within modern production systems. As the industry evolves, new challenges consistently emerge. In this review, we summarize prevalent welfare-related behaviors on pig farms and the latest research on the role of the MGBA in influencing behaviors, providing theoretical foundations and potential strategies for improving pig welfare.

## 2. Material and Methods

A comprehensive literature search was conducted using Google Scholar and PubMed databases with the following keywords: “pig”, “microbiota”, “behavior”, “welfare”, “gut”, “brain”, “microbiota–gut–brain axis”, “environment”, “disease”, “immune”, “endocrine”, “neural”, “tail-biting”, “fecal microbiota transplantation (FMT)”, “probiotic”, “prebiotic”, “fiber”, “vitamin”, “amino acid”, and “mineral.” Only studies relevant to the themes of “gut microbiota and welfare”, “gut microbiota and pigs”, “gut microbiota, pigs, and behavior”, “gut microbiota and behavior”, and the “microbiota–gut–brain axis” were included in this review. The initial search was conducted between June and October 2024 and was updated in April 2025. Additionally, relevant references cited within the selected studies were also screened and included where appropriate.

## 3. Association Between Pig Welfare and Behavior

### 3.1. Freedom from Hunger and Thirst

The first freedom is to provide animals with adequate clean water and the food necessary to maintain health and energy, ensuring that they are free from hunger and thirst. Depending on the age group and market demands, pigs are either fed on a fixed schedule with measured portions or allowed to feed ad libitum. This approach ensures that pigs are not hungry while minimizing feed waste. However, pregnant sows are often subjected to restricted feeding to prevent excessive weight gain. Under these conditions, sows may exhibit abnormal behaviors such as bar chewing or oral stereotypies, which can be alleviated by increasing the fiber content in their diet to enhance satiety [16]. Under normal circumstances, pigs are able to drink freely, but they also need to ensure that the water is clean and provided at an appropriate temperature. Inadequate nutrition can lead to the emergence of abnormal behaviors in pigs. As omnivorous animals, pigs in the wild would search for a variety of foods to satisfy their hunger [17]. However, when kept in confinement, their diet is strictly controlled, which may result in an insufficient supply of essential nutrients, such as proteins, minerals, and vitamins, thereby contributing to the development of abnormal behaviors. Mineral deficiencies may prompt pigs to wall-chewing or drinking contaminated water, including feces-contaminated water, in an attempt to compensate. Furthermore, a deficiency in phosphorus can lead to lameness in fattening pigs [18]. Additionally, insufficient tryptophan intake has been associated with aggressive and abnormal behaviors [19].

### 3.2. Freedom from Discomfort

The second freedom is to provide animals with suitable housing or resting areas that allow for comfortable rest and sleep, ensuring that they are free from discomfort. The housing system should not only guarantee adequate space and comfort but also take into account the need for social interaction among animals, enabling them to fully express their natural behaviors. Stocking density has multiple effects on pigs, influencing the metabolism, gut morphology, and immune function of growing pigs [20]. It also alters their behaviors, including feeding, movement, and lying down [21]. Appropriate environmental temperatures are also part of ensuring pig welfare. When the temperature is too high, animals may experience heat stress, a form of pressure that increases the need for heat dissipation [22]. To cope with this stress, animals adjust their behaviors. High temperatures not only cause discomfort from hunger and thirst, but if pigs are unable to move to a cooler area to rest, they may become irritable and more aggressive [23]. Additionally, pigs that feel uncomfortable due to overheating or poor air circulation may become agitated, leading to increased aggression. However, the changes in lying behavior vary among species. While high temperatures reduce lying time in dairy cows, pigs tend to increase lying time to enhance heat dissipation through increased contact between their skin and the floor [24]. Low temperatures also impact pig welfare, particularly in piglets, as they can reduce growth performance and nutrient digestibility, and in severe cases, lead to diarrhea [25]. In cold environments, pigs typically huddle together, reducing movement such as standing to conserve body heat. Overall, maintaining an optimal temperature range is essential for promoting the well-being and productivity of pigs in a farming environment. Furthermore, maintaining cleanliness in living environments is crucial. Timely cleaning of pens helps reduce the spread of diseases and provides pigs with the freedom to lie down comfortably. Therefore, the proper management of pig housing space is essential not only for ensuring adequate movement space but also for safeguarding their overall health.

### 3.3. Freedom from Pain, Injury, and Disease

The third freedom is to ensure proper immunization for animals, prevent diseases, and provide timely treatment for sick animals, thereby protecting them from pain, injury, and illness. Behavioral changes due to illness are often first noticed by farmers and passed along informally, which has resulted in limited and relatively outdated documentation in the literature. Diseases can lead to changes in behavior and mental state, making behavioral alterations useful indicators for identifying the onset of illness. Simple changes in feeding and drinking habits may be indicative of infectious diseases, such as pneumonia outbreaks or Salmonella infections [26]. More specific abnormal behaviors, such as walking while shaking the ears, may suggest the presence of otitis media. Drinking contaminated water could indicate the presence of parasites, which compete with the pig for nutrients, leading to deficiencies. Additionally, behaviors such as isolating from the group and lying down, or lying in water, may signal gastrointestinal issues or fever. Lameness in pigs is often associated with leg-related conditions, such as osteochondrosis, arthritis, or hoof diseases [27].

### 3.4. Freedom from Fear and Distress

The fourth freedom is to ensure that animals are provided with appropriate conditions and handling, protecting them from fear and mental distress. When pigs experience fear, they tend to become more tired, with the most common manifestations being increased lying down, reduced walking, and decreased exploratory behavior. A distinctive sign is a lower degree of tail curling, often accompanied by a drooping tail. In contrast, when a group is startled, the opposite behavior is observed, with heightened group agitation. Emotions are also conveyed through sight, smell, and sound, with vocalizations being a significant tool for assessing the emotional aspects of animal welfare. After weaning, piglets emit high-frequency vocalizations [28]. Additionally, these high-frequency sounds can be used to detect and monitor ear-biting behavior. Post-weaning, piglets emit high-frequency screams, which can be employed to detect and monitor ear-biting incidents. In contrast, low-frequency vocalizations are considered positive indicators of welfare [29].

### 3.5. Freedom to Express Normal Behavior

The fifth freedom requires several prerequisites, including providing animals with sufficient space, appropriate facilities, and the company of others, allowing them to express their natural behaviors freely. Exploration and play are innate behaviors in pigs, and when these behaviors are observed, it indicates that the pigs enjoy relatively good welfare [30]. To support these behaviors, pigs should be provided with materials such as straw and branches for play, as well as substances that are chewable, ingestible, and manipulable for exploration. Pregnant sows possess an innate urge to construct nests. Therefore, housing sows in pens with straw, and ensuring adequate space and materials, allows their natural nesting behavior to be expressed and improves their welfare.

When pigs are unable to express their natural behaviors, noticeable changes in behavior can occur. A lack of opportunities for environmental exploration may lead to increased aggression, TB, and the emergence of stereotypic behaviors [31]. Environmental enrichment can fulfill the exploratory needs of pigs, leading to more positive behaviors and improved human–animal interactions. Moreover, when exploratory behaviors are satisfied, social interactions tend to increase while lying behavior decreases [21]. In summary, monitoring the behavior of pigs offers significant understandings regarding their physiological and psychological conditions, thereby enabling the implementation of timely interventions to enhance the welfare of pigs.

## 4. Gut Microbes Influence Pig Behavior

### 4.1. Main Microbes in the Gastrointestinal Tract of Pigs

Gut microbiota have been shown to contribute to neuroregulation, thereby influencing behavior [32]. To better understand the influence of gut microbes on behavior, the core microbes in the pig gut were first analyzed. Extensive research has revealed that Firmicutes and Bacteroidetes are the most abundant phyla of pig gut microbiota, which may also include Proteobacteria [33,34,35,36]. The core genus of bacteria considered in different studies varies, and for this reason, 20 articles examining the gut microbiota of pigs of different breeds, ages and sexes were collected to summarize the core genus. Out of these, 3 articles were review articles and the remaining 17 were research articles, the results of which were summarized in tabular form (Table 1).

**Figure 2:**
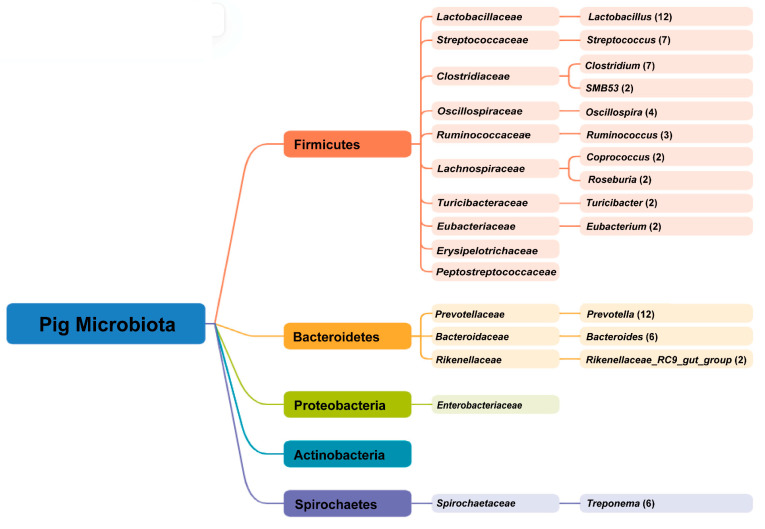
Main microbes in the gastrointestinal tract of pigs.

A meta-analysis of 20 publicly available datasets revealed that no single bacterial genus was universally detected across all samples [37]. However, eight core genera *Clostridium*, *Blautia*, *Lactobacillus*, *Prevotella*, *Ruminococcus*, *Roseburia*, the RC9 gut group, and *Subdoligranulum* were present in over 90% of the samples. Notably, *Prevotella* exhibited the highest relative abundance among all detected microbiota. Another study synthesized results from multiple published works to characterize the composition and dynamic change of the gut microbiota across different age stages [38]. The analysis revealed a shift in dominant bacterial genera around the weaning period. However, three core genera, *Bacteroides*, *Prevotella*, and *Lactobacillus*, were consistently detected regardless of age. Furthermore, a comprehensive analysis encompassing 3313 fecal microbial communities from 349 pigs across 14 published studies identified ten bacterial genera consistently present in all four stage-specific core microbial groups, including *Lactobacillus*, *Parabacteroides*, *Desulfovibrio*, *Blautia*, and *Lachnoclostridium* [39]. Through systematic evaluation of multiple studies examining gut microbiota, we identified 14 microbial genera that consistently appeared as core components across at least two independent investigations, which were *Prevotella*, *Lactobacillus*, *Streptococcus*, *Clostridium*, *Bacteroides*, *Treponema*, *Ruminococcus*, *Oscillospira*, *Coprococcus*, *SMB53*, *Roseburia*, *Turicibacter*, *Eubacterium and Rikenellaceae_RC9_gut_group* (Figure 2).

**Table 1:** Dominant genus in 17 research articles.

Research Subject	Dominant Genus	Refference
Fecal samples collected from 287 pigs of 17 breeds	*Prevotella*, *Bacteroides*, *Clostridium*, *Ruminococcus* and *Eubacterium*	[40]
Fecal samples collected from 244 Bamaxiang pigs and cecum content samples from 256 Erhualian pigs	*Treponema*, *Rikenellaceae_RC9_gut_group*, *Prevotellaceae_NK3B31_group*, and *Prevotella*	[41]
Rectum contents of 288 Duroc × Iberian pigs	*Prevotella* and *Treponema*	[42]
Rectum contents of 102 Diannan small-ear pigs	*Streptococcus*, *Lachnospiraceae*, *Oscillospiraceae_UCG-002*, and *Lachnospiraceae_XPB1014_group*	[36]
Fecal samples of 120 pigs within different breeds	*Lactobacillus*, *Prevotella*, *Treponema*, *Oscillospira*, *Clostridium*, *Ruminococcus*, *Holdemania*, *Streptococcus*, *Bacteroides* and *Coprococcus*	[33]
Gastrointestinal tract samples of 13 Iberian pigs	*Lactobacillus* and *Clostridium*	[43]
Fecal samples of 11 Tibetan pigs	*Prevotella* and *Treponema*	[44]
Fecal samples of 105 Jinhua pigs	*Lactobacillus*, *Bifidobacterium*, *Streptococcus*, *Prevotella*, *Bacteroides*, *Turicibacter*, *Clostridium*, *Treponema*, *Allobaculum*, and *SMB53*	[45]
Fresh fecal samples of 15 Duroc × Landrace × Yorkshine pigs	*Lactobacillus*, *Streptococcus*, *SMB53*, *Oscillospira*, and *Prevotella*	[46]
Fecal samples 8 Ningxiang pigs	*Lactobacillus*	[47]
Fresh fecal samples of Duroc (n = 505), Landrace (n = 2120) and Yorkshire (n = 3754)	*Streptococcus* and *Lactobacillus*	[48]
Rectal content of 288 Duroc Iberian pigs	*Rikenellaceae RC9 gut group*, *Treponema*, *Prevotella*, and *Muribaculaceae genus*	[49]
Fecal samples of 10 Yorkshire-Landrace pigs	*Clostridium*, *Escherichia*, *Megasphaera* and *Lactobacillus*	[50]
Fecal samples of 274 Suhuai pigs	*Lactobacillus*, *Clostridium*, *Streptococcus*, *unclassified p Firmicutes*, *Prevotella*, *unclassified f Lachnospiraceae*, *Bacteroides*, *Ruminococcus*, *Eubacterium*, and *Roseburia*	[51]
Gastrointestinal tract and fecal samples of 6 Landrace × Large White pigs	*Lactobacillus*, *Actinobacillus*, *Romboutsia*, *Escherichia-Shigella*, *Terripsorobacter*, and *Campylobacter*	[52]
Fecal samples of 518 piglets	*Prevotella* and *Roseburia*	[53]
Fecal samples of Duroc (n = 6), Hampshire (n = 4), Landrace (n = 7), Yorkshir (n = 6)	*Streptococcus*, *Prevotella Lactobacillus*, *Clostridium Turicibacter*, *Oscillospira*, *Coprococcus*, *Bacteroides* and *Ruminococcus*	[54]

Although they are all considered core microbiota, their roles vary. The relative abundance of *Firmicutes* is higher in the TB pigs, including families such as *Lachnospiraceae*, *Ruminococcaceae*, and *Clostridiales Family XIII* [55]. Both the tail-biters and the bitten pigs exhibit lower relative abundance of *Lactobacillus*, which is related to higher psychological stress [56]. *Lactobacillus* also produces GABA, which can promote feed intake in pigs. Both *Clostridium* and *Lactobacillus* have been shown to be linked to higher feed efficiency, though the relationship between *Prevotella* and feed efficiency remains unclear. Some studies suggest a negative correlation, while others indicate a positive correlation [57]. However, pigs with *Prevotella*-dominant microbiota tend to have higher feed intake and spend more time at the feeding trough, *Ruminococcaceae* also plays a similar role [58,59]. Additionally, *Prevotella* has a negative correlation with exploratory behavior. In rodents and humans, *Prevotella* has been associated with depression or anxiety, suggesting that the reduction in exploratory behavior may indicate a degree of depression in pigs [60].

### 4.2. Microbiota and Pig Behavior

Behavior, as a direct manifestation of physiological states, is inevitably closely linked to the microbiota. Pig behavior can be categorized into ten major types: feeding behavior, excretion behavior, group living behavior, fighting behavior, sexual behavior, maternal behavior, activity and sleeping behavior, exploratory behavior, abnormal behavior, and after-effect behavior. Some of these behavioral activities are influenced by the gut microbiota (Table 2).

#### 4.2.1. Feeding Behavior

The food consumed influences the gut microbiota, which in turn affects food intake. Feed intake in pigs is associated with colonic carbon substrates. The relative abundance of primary degrader Ruminococcaceae was dynamically correlated with carbon substrate levels. In contrast, the fluctuations of secondary degraders, including Lactobacillaceae and Streptococcaceae, were highly correlated with sugar metabolites. These sugar metabolites exhibit oscillations with microbial metabolites, including acetate, propionate, butyrate, and valerate, within defined time shifts [66]. He et al. conducted a comprehensive study monitoring the feeding behavior of three different pig breeds across various growth stages and correlating this behavior with gut microbiota [59]. The results indicated that *Marvinbryantia*, *Dorea*, *Blautia*, and *Ruminococcaceae_UCG-014* exhibited a positively correlation with feeding, and interestingly, these microbiota have been inversely associated with depression or anxiety in mice and human models, leading to the speculation that a positive mood state might enhance feeding. Conversely, certain members of the *Ruminococcaceae* family, namely *Christensenellaceae*, *Family_XIII_AD3011_group*, and *Christensenellaceae_R-7_group*, along with *Ruminococcaceae_UCG-004*, demonstrated a negative correlation with feed intake. Notably, *Christensenellaceae_R-7_group* and *Ruminococcaceae_UCG-004* are capable of producing SCFAs, and an elevation in SCFA levels has been shown to suppress appetite and subsequently foraging behavior. Additionally, this study uncovered both positive and negative correlations between *Ruminococcaceae* and foraging behavior, suggesting that diverse members within the *Ruminococcaceae* family possess distinct regulatory mechanisms for foraging activities.

#### 4.2.2. Excretion Behavior

Excretory behavior must be associated with the microbiota of the gut, especially a particular excretion, diarrhea. Diarrhea in newborn piglets has been associated with an increase in the relative abundance of *Clostridiaceae*, *Campylobacter*, and *Sutterella*, as well as the *Prevotella* [61]. The increase in relative abundance of *Prevotella* spp. was related to a decrease in *E. coli* and most of the beneficial bacteria being classified as Firmicutes (e.g., *Blautia*, *Clostridium*, *Lactobacillus*, *Streptococcus*, and *Enterococcus*). Piglets exhibit varying degrees of adaptability to weaning. Those with a higher daily weight gain possess gut microbiota that approach maturity by the time of weaning, whereas in piglets with a lower daily weight gain, the gut microbiota remain in a state of development [67]. Notably, the relative abundances of *Anaerotruncus* and *Bacteroides*, both recognized as pathogenic bacteria, are elevated in the intestines of lighter-weight piglets, potentially predisposing them to diarrhea and even more severe intestinal disorders [68]. Microbiota can regulate the synthesis of 5-HT, and both 5-HT and its receptors are located on the neurons between the muscle layers, mediating the release of excitatory neurotransmitters [13]. This, in turn, promotes the contraction or relaxation of smooth muscles, as well as intestinal secretion. Therefore, excretion is also influenced by the gut microbiota.

#### 4.2.3. Group Living Behavior

Pigs are herd animals and they set up hierarchies for the purpose of optimizing resource utilization and decreasing the occurrence of conflicts. There are differences in gut microbiota across different hierarchies [62]. *Faecalibacterium*, *Peptococcus*, and *Oliverpabstia* are more abundant in dominant pigs, while *Holdemanella*, an indicator of pig stress, is more prevalent in subordinate ones. Therefore, there is a correlation between group living behavior and MGBA.

#### 4.2.4. Fighting Behavior

The neural pathways controlling struggling behavior include the frontal cortex, amygdala, and hypothalamus and are associated with serotonergic and dopaminergic systems. Piglets were prone to aggressive behavior during weaning mixing [69]. When piglets are weaned and grouped together, they tend to exhibit aggressive behaviors. Piglets that are more prone to aggression have lower levels of blood 5-HT and dopamine in their plasma. Moreover, before being mixed, aggressive pigs having higher levels of either plasma tryptophan or platelet count and lower platelet 5-HT content are more likely to show increased aggression after grouping. Enterochromaffin cells synthesize and secrete most of 5-HT, which is regulated by microbiota and stored by platelets and released when needed, so it is possible to link the MGBA to fighting behavior [13,70].

#### 4.2.5. Sexual Behavior

Research has found that gut microbiota affect the biosynthesis and metabolism of sex hormones, subsequently influencing sexual behavior. Puberty is a crucial stage for reserve sows and is typically modulated by sex hormones. Reserve sows that experience normal puberty exhibit a high prevalence of *Prevotella*, *Treponema*, and *Faecalibacterium*, whereas *Lachnospiraceae*, *Ruminococcus*, *Coprococcus*, and *Oscillospira* are relatively more abundant in those with failed puberty [63]. Wang et al. determined that *Bacteroidia*, *Lactobacillaceae*, and *Lactobacillus* are related to the resumption of normal estrus in sows. In contrast, *Actinobacteria*, *Ruminococcus*, *Clostridium*, *Streptococcus*, *Streptococcaceae*, *Lachnospiraceae*, and *Clostridiales* are more prevalent with sows that inability to come into estrus normally again [64]. It can further be hypothesized that the intestinal microbiota regulate estrus in sows by modulating metabolites related to steroid synthesis or by influencing the action of sex hormones. It was also observed that reserve sows with failed puberty had weaker retinol metabolism. Retinol is involved in the formation of steroid hormones, and estrogen in the somatic circulation promotes gonadotropin-releasing hormone in the hypothalamus, which further triggers estrus. Through the Spearman analysis, it was found that *Prevotella* is associated with retinol metabolism, leading to the speculation that it promotes hormone production and oocyte maturation to initiate estrus.

#### 4.2.6. Exploratory Behavior

Exploratory behavior is also influenced by the microbiota. In the open field test, the distress and anxiety screams of piglets had a positive relation with the abundances of *Coprococcus 3*, *Eubacterium*, and *Coprostanoligenes* in the intestine [60]. Conversely, during novelty exploration, exploratory behavior was negatively associated with the abundances of *Prevotellaceae NK3B31* groups and *Prevotella 9*, but positively correlated with microbiota like *UBA1819* and *Atopobium*. Notably, *Prevotellaceae NK3B31* was positively related to salivary cortisol [71]. Piglets supplemented with synbiotics adapted earlier to novelty exposure [65].

#### 4.2.7. Abnormal Behavior

Abnormal behavior refers to a type of repetitive, purposeless behavior that occurs when psychological or physiological needs are unmet, often leading to self-harm or harm to others. It is an external manifestation of the inability of animal to adapt to overcrowded and impoverished living conditions and is quite common in modern livestock farming. In pigs, the most common form of abnormal behavior is TB. Gut microbiota primarily influence abnormal behavior through metabolic products and by regulating neurotransmitters. Pigs administered antibiotics exhibit a heightened susceptibility to tail injuries and display more ear-biting and aggressive behaviors in contrast to those without antibiotic supplementation, although the specific gut microbiota involved remain unidentified [72]. TB and bitten pigs experience elevated levels of anxiety compared to unaffected pigs, with this anxiety being modulated by the gut microbiota [73]. The relative abundance of Firmicutes is greater in TB pigs, which is driven by the increased presence of *Lachnospiraceae*, *Ruminococcaceae*, and *Clostridiales Family XIII* [55]. Clostridiales have been linked to diverse behavioral disorders, and the *Ruminococcaceae* and *Lachnospiraceae* families can influence 5-HT biosynthesis, consistent with the higher 5-HT levels in the prefrontal cortex (PFC) of TB pigs. The enhanced conversion of 5-HT to 5-HIAA in the PFC of TB pigs was also observed [74].

Regarding microbial metabolites, SCFAs were investigated and found to be decreased in the feces of biting pigs and in the circulation of victim pigs, yet this finding conflicts with the microbial data, indicating that the relation between microbiota as well as their metabolites and TB behavior necessitates more comprehensive exploration. In another study, the relative abundance of *Lactobacillus* in TB and bitten pigs was lower than that in normal pigs [56]. *Lactobacillus* is a probiotic that has been widely used and studies have found that low relative abundance is associated with high stress as well as the development of manipulative behavior in pigs [75]. Consequently, the intake of *Lactobacillus* can effectively improve stress-induced corticosterone, depression- and anxiety-related behaviors, primarily mediated by modulation of GABA mRNA expression levels in the brain and the involvement of the vagus nerve [76]. While it is established that TB is associated with gut microbiota, the relationship and mechanisms of influence require further in-depth investigation.

A range of abnormal behaviors, including belly nosing, fence-biting, and ear-biting, can be alleviated by improving the environment and increasing the number of objects in the environment that can be explored or played with. Nevertheless, environmental enrichment impacts gut microbiota, leading to a decline in Enterococcus and an increase in *Ruminiclostridium_9*, *Ruminococcus gauvreauii_group*, *Christensenellaceae_R_7_group*, and *Prevotella_2*, which can influence the production of SCFAs. Moreover, while environmental enrichment reduces and alleviates pig stress, the precise mechanism of action and its correlation with gut microbiota have not been determined [77].

#### 4.2.8. After-Effect Behavior

After-effect behavior refers to the process through which pigs gradually become familiar with new stimuli after birth, which is closely related to memory and cognition. Among the behavioral alterations in piglets following prebiotic supplementation, only the fence-barrier task exhibited a significant correlation with several microbiota. *Bacteroidetes* and *Prevotella* were inversely related to the time taken to successfully cross the fence barrier, while Firmicutes (*Ruminococcaceae*, *Faecalicoccus*, and *Clostridium XIVa* and *XVIII*) showed a positive correlation [65]. However, in human studies, *Prevotella* was more abundant in cognitively impaired individuals, and a reduction in *Ruminococcaceae* was associated with cognitive impairment [78,79].

Although there is sufficient evidence to support a correlation between gut microbiota and behavior, the specific mechanisms by which gut microbiota regulate behavioral changes in pigs remain poorly understood. Future research should focus on the roles of the immune, endocrine, and neural pathways to clarify how gut microbiota influence behavior. This will help identify potential targets for microbial intervention and contribute to improving pig welfare.

## 5. The Role of Microbiota in Regulating Behavior

**Figure 3:**
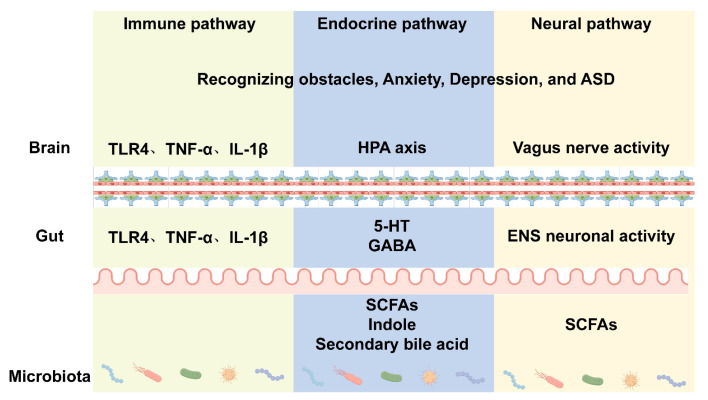
The role of microbiota in regulating behavior.

Behavior is regulated by the nervous system, and the gut–brain axis connects the central nervous system to the gastrointestinal tract, forming a bidirectional communication system in which microbes are a critical component [80]. Gut microbiota regulate behavior through three main pathways: (1) the immune pathway, which influences the neuroimmune response; (2) the endocrine pathway, through which gut microbiota regulate gut endocrine cells as well as the hypothalamic–pituitary–adrenal (HPA) axis, thus influencing neurotransmitter synthesis; and (3) the neural pathway [81] (Figure 3).

### 5.1. Immune Pathway

Gut microbiota can modulate the host’s immune responses, and the interaction between microbiota and the immune system is considered a contributing factor in the pathogenesis of neuroinflammation and neuropsychiatric disorders. These microbiota–host interactions are mediated through the release of cytokines and chemokines, which circulate via the blood and lymphatic systems, thereby influencing immune signaling throughout the body. Intestinal inflammation can be induced by imbalances in the gut microbiota, which activates immune cells and stimulates the release of pro-inflammatory cytokines. These cytokines can enter the brain via systemic circulation, contributing to neuroinflammation and altering neural plasticity, which in turn affects cognition, emotion, and behavior. High-fat diets (HFDs) have been shown to alter the gut microbiota, increasing levels of TLR4, IL-6, IL-1β, and TNF-α in the colon, thereby inducing intestinal inflammation and impairing cognitive performance in mice [82]. HFD-induced microbial alterations also elevate hippocampal and PFC levels of IL-1β, TNF-α, p-NF-κB, p-IκB-α, MyD88, and TLR4, leading to neuroinflammation and the development of anxiety- and depression-like behaviors [83]. Translocation of bacterial lipopolysaccharides (LPS) in the gut activates the LPS/TLR4 signaling pathway, resulting in elevated pro-inflammatory cytokine levels in the brain [84]. These cytokines can cross the BBB, trigger stress responses, and ultimately result in neuroinflammation and cognitive disorders.

In addition, gut microbiota can regulate the gut–brain axis by regulating cytokine production from immune cells. Dimethyl itaconate has been shown to modify the structure of gut microbiota, thereby reducing hippocampal levels of pro-inflammatory cytokines like IL-6, IL-1β, and TNF-α, weakening HFD–induced neuroinflammation, and enhancing synaptic protein expression to improve cognition [85]. Probiotics exert neuroprotective effects by modifying gut microbiota to reduce LPS production, thereby inhibiting the TLR4/NF-κB signaling pathway, alleviating BBB damage and neuroinflammation, and improving neuronal injury and memory deficits in mice [86]. *Clostridium butyricum* elevates the concentration of butyrate in the gut, which inhibits the increase in IL-1β and TNF-α in the brain and protects against cognitive impairment [87]. FMT has verified that alterations in microbiota can improve cognition. Fungi in the gut mucosa can increase IL-17 in the gut and act on cortical neurons, promoting social behaviors in mice [88].

Due to the presence of the BBB, circulating B cells and T lymphocytes are restricted from entering the underlying parenchyma. Therefore, immune regulation within the CNS is primarily mediated by microglia, astrocytes, and oligodendrocytes. Gut microbiota can also influence the development and function of CNS-resident cells, thereby modulating the gut–brain axis. In germ-free (GF) mice, microglia have impaired maturation, altered cell proportions, and an immature phenotype, resulting in compromised innate immune responses [89]. On the other hand, the microbial metabolites, SCFAs, have been shown to restore microglial homeostasis, highlighting the function of microbiota in modulating glial cell. FMT can reduce the activation of astrocytes and microglia in the substantia nigra, alleviating neuroinflammation and exerting neuroprotective effects [90]. Furthermore, microbial metabolism of tryptophan produces aryl hydrocarbon receptor (AHR) agonists that regulate AHR activity in astrocytes, influencing CNS inflammation [91]. Disorder of the gut microbiota can activate complement component C3, resulting in abnormal synaptic pruning mediated by microglia [92]. *Roseburia intestinalis* has been shown to modulate microglial and astrocytic activity, decreasing quinolinic acid levels and increasing kynurenic acid levels, thereby protecting against synaptic loss and reducing depression- and anxiety-like behaviors [7].

### 5.2. Endocrine Pathway

Enteroendocrine cells can be stimulated to secrete hormones by gut microbiota, which also modulate the stress response via the HPA axis. Acute stress induces alterations in serotonergic neurotransmission in the PFC and hippocampus, contributing to behavioral disorders such as depressive disorder [12]. Enterochromaffin cells, located in the gastrointestinal epithelium, synthesize most of 5-HT [70]. The production and secretion of 5-HT are regulated by various microbial metabolites, including indoles, secondary bile acids, and SCFAs. *Lactobacillus amylovorus* is positively correlated with 5-HT production in the pig intestine [93]. When 5-HT levels are sufficient, it promotes gastrointestinal motility by decreasing gastric acid secretion and increasing small intestinal peristalsis [94]. In contrast, 5-HT deficiency may result in anorexia and aggressive seizure activity. Although peripheral 5-HT cannot cross the BBB, serotonergic signaling in the brain can still be significantly influenced by the gut microbiota through activation of stress–response systems such as the HPA axis [13].

The neuroendocrine system serves as the intersection between the CNS and endocrine glands, responsible for regulating and balancing the secretion and function of hormones. The HPA axis is the primary neuroendocrine system regulating various physiological processes [95]. Many mood-related disorders are associated with abnormalities in the HPA axis, with depression being one of them. Studies have shown that maternal dietary habits, such as HFD, can alter the gut microbiota in offspring, leading to abnormal HPA axis development and affecting their stress response [96]. Chronic restraint stress experiments have demonstrated that compared to mice with a normal gut microbiota, GF mice exhibit increased resilience to anxiety and their HPA axis is overactive [97]. This suggests that gut microbiota dysbiosis can impact the HPA axis, leading to mood-related disorders. Furthermore, probiotics can modulate the HPA axis to cope with stress. During stress, the abundance of Proteobacteria in the gut increases, which activates the NF-κB pathway via the HPA axis, resulting in anxiety. However, oral administration of commensal *lactobacilli* can alleviate this effect [98].

Furthermore, microbiota can affect the synthesis of neurotransmitters. Increased 5-HT metabolism (elevated 5-HIAA levels) was also examined in the prefrontal cortex of tail-biting pigs, which correlated with a higher relative abundance of *Ruminococcaceae* in the gut [55]. Disturbance of pig gut microbiota by antibiotics leads to significant reductions in GABA, dopamine, 5-HT and brain-derived neurotrophic factor concentrations in the hypothalamus [99]. Cecal starch infusion decreased *Ruminalococcus*, *Megalococcus*, *Synechococcus*, *Olsonella*, *Streptococcus*, and *Lactobacillus*, whereas *Rikenellaceae RC9*, *Erysipelotrichaceae unclassified*, *Ruminalococcus UCG-005*, and *Ruminalococcus UCG-002* increased, and 5-HT, dopamine and brain-derived neurotrophic factor also increased in the hypothalamus [100]. It may be that microbiota can alter the expression of genes involved in neurotransmission [101]. In GF mice, a lack of microbiota was found to reduce the levels of 5-HT and GABA in the gut. Under stress and anxiety conditions, GABA synthesis decreases, which correlates with a reduced abundance of *Bacteroides eggerthii* [102]. GABA mRNA expression in the hippocampus was altered after administration of *Lactobacillus rhamnosus* JB-1, thereby alleviating depression and anxiety induced by stress [76]. Oral administration of GABA also relieves stress and reduces anxiety [103]. Additionally, GABA can affect the release of other neurotransmitters, exerting an antidepressant effect [104].

### 5.3. Neural Pathway

Neuronal pathways are composed of enteric nervous system (ENS) and extrinsic neurons (the vagus nerve). The interface between the microbiota and enterocytes is the ENS, responding to microbiota and their metabolites. The communication of the ENS relies on the pathway from intestinal neurons to sympathetic ganglia and finally to primary afferent neurons [105]. Gut microbiota also mediate the development and some functions of the ENS. GF mice exhibit morphological defects in the ENS and impairments in excitatory transmission, and these defects can be reversed by microbiota colonization [106]. Microbial metabolites can also affect the ENS. Mice whose gut microbiota have been cleared by antibiotic treatment show enhanced neuronal survival after LPS treatment, while SCFAs can directly restore the number of neurons, which means they can stimulate enteric neurogenesis [107]. *Lactobacillus reuteri* can activate the potassium channels that depend on calcium in the ENS, enhancing neuronal excitability, which may further directly mediate intestinal motility and pain perception [108].

The vagus nerve, the tenth cranial nerve, is a crucial neuron facilitating bidirectional communication between the gut and the brain. It is influenced by the digestive environment and the composition of the gut microbiota, and can transmit information regarding gastrointestinal motility, secretion, absorption, and epithelial permeability [109]. The microbiota regulates sympathetic neurons through the MGBA [110]. Depletion of the microbiota leads to an increase in the expression of *cFos*, which is a neuronal transcription, thereby activating neurons. Once bacteria capable of producing SCFAs are colonized in GF mice, the expression of *cFos* is decreased. The vagus nerve represents a vital component within the MGBA, with approximately 80% of it conveying messages between the gut and the brain [111]. It mediates the impacts of gut microbiota on 5-HT and dopamine neurotransmission in the brain, as well as on emotional and social behaviors related to mood disorders and neurodevelopment [76,112,113]. *L. reuteri* has been shown to alleviate social disorder in autism spectrum disorder (ASD) mice. However, when the vagus nerve is severed, *L. reuteri* ceases to function [114]. Similarly, *Lactobacillus rhamnosus* (JB-1) reduces anxiety-related behaviors and induces changes in GABA receptor expression, but these neurochemical and behavioral changes do not occur in mice with vagus nerve transection [115].

Current research on the MGBA is predominantly based on rodent models, reflecting the fact that investigations are still focused on fundamental mechanisms. However, to improve the translational relevance of MGBA studies to humans, future research should incorporate more pig-based models, as the pig’s MGBA shares greater anatomical and physiological similarities with that of humans [73,116].

## 6. Approaches for Targeted Regulation of Gut Microbiota to Promote Welfare

**Figure 4:**
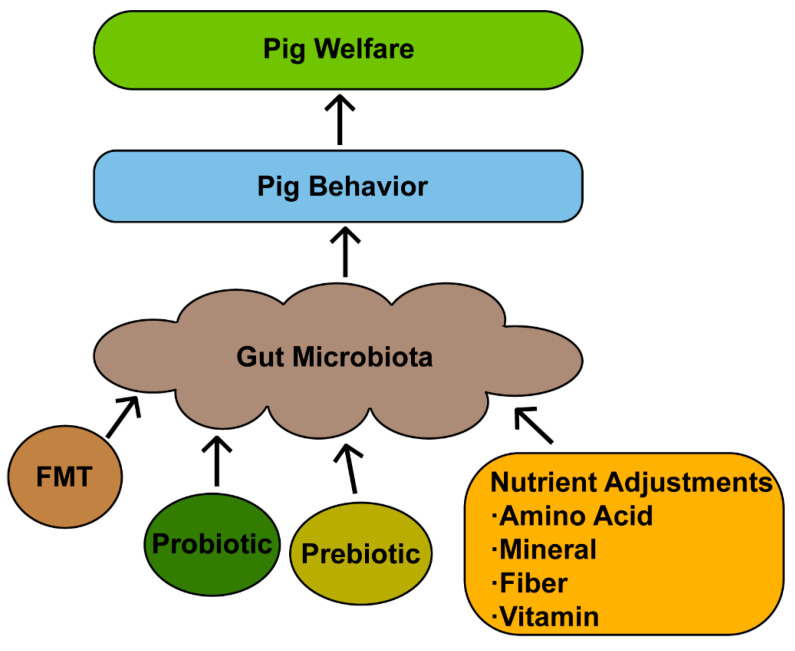
The strategies for promoting pig welfare via gut microbiota.

Given the close relationship between microbiota and behavior, targeted regulation of the gut microbiota has emerged as a promising method of improving pig welfare, commonly including direct modulation of gut microbiota through FMT, probiotics, and prebiotics, and indirect action through nutrient adjustments (Figure 4).

### 6.1. Fecal Microbiota Transplantation

FMT is a method that transfers fecal matter to an individual, directly altering gut microbiota and reconstructing the gut microbiota, subsequently influencing behavior. When feces from a healthy population are transplanted into mice displaying anxiety-like and depressive-like behaviors, it suppresses the kynurenine pathway metabolism by modulating the rate-limiting enzyme and augments 5-HT levels in the frontal cortex and feces [7]. In the case of feather-pecking behavior, which is recognized to be associated with gut microbiota, chicks that received fecal transplants in the initial two weeks of life and homologous microbiota transplants (i.e., from their strain) exhibited more positive behavioral responses [117]. Although currently there are no investigations concerning FMT and swine behavior, extensive research has uncovered benefits of FMT in pigs, such as enhancing feed efficiency, improving the gut barrier, and diminishing weaning diarrhea [118,119,120]. This implies that FMT could be advantageous for pigs and further leads to the speculation that it might be employed to alleviate abnormal and struggling behaviors in pigs.

### 6.2. Probiotic

Probiotics are commonly used to regulate gut dysbiosis. Recent research has indicated that probiotics can enhance dendritic arborization, increase spine density, and normalize oxytocin levels, thereby ameliorating ASD-related behaviors [121]. Moreover, probiotics reduce neuroinflammation by suppressing pro-inflammatory microglia activation [122]. There is also evidence suggesting that probiotics might alleviate stress-, depression-, and anxiety-related behaviors through enhancing the effects of 5-HT and GABA [123,124]. *Bacillus*, *Lactobacillus*, *Bifidobacterium*, *Enterococcus*, and *Saccharomyces* are prevalently utilized probiotics within the swine industry, being widely applied across diverse swine production stages to modulate gut microbiota, enhance growth performance, fortify the intestinal barrier, mitigate diarrhea, and diminish uterine and/or udder diseases. Nevertheless, research pertaining to the improvement of animal welfare, particularly in relation to behavioral regulation, remains unclear.

Behavior changes when pigs are sick; for example, pigs with Salmonella infection tend to lie down more and eat less. However, with the administration of *Bacillus licheniformis*, these behaviors improved and exploratory behaviors increased [125]. Lactobacillus has been found to reduce piglets’ alertness to auditory threats and modify anxiety-like states [126]. Given the common practice of supplementing *Lactobacillus* or other beneficial microbiota before and after weaning to prevent health issues and enhance pig production, early-life administration of *Lactobacillus* may prove to be a valuable means of enhancing the welfare of intensively farmed pigs. Pregnant sows provided with a probiotic complex of *Lactobacillus*, *Bifidobacterium*, *Enterococcus*, and *Streptococcus* exhibited not only reduced fear and lower cortisol levels but also less aggressive behavior in their offspring [127]. The supplementation of BAYKAL EM-1 efficaciously alleviates restlessness in weaned piglets, potentially attributable to the capacity of probiotics to modify hypothalamic neurotransmitters [128,129].

### 6.3. Prebiotic

Organic substances cannot be digested or absorbed by the host but have the ability to improve the proliferation and metabolism of beneficial bacteria in the body selectively are prebiotics, which improve host health. Compared to probiotics, which may lose their efficacy during production and usage, prebiotics are more stable and relatively cost-effective. However, the application of prebiotics in enhancing welfare is still relatively less common.

Current research on the effects of prebiotics on pig behavior primarily focuses on cognitive outcomes, especially memory. Studies show that supplementation with 2′-fucosyllactose alone has limited effects on memory and exploratory behavior but increases the relative volume of the pons in piglets [130]. In contrast, the addition of oligofructose has been shown to enhance cognitive performance and modulate hippocampal gene expression [131]. A combined supplementation of polydextrose and galactooligosaccharides not only improved memory performance but also promoted more adaptive exploratory behavior, such as increased willingness to try novel foods and decreased interest in familiar objects [132]. Meanwhile, the volatile fatty acids in the intestine decrease, while butyric acid shows an increasing trend in the blood and a decreasing trend in the hippocampus. Interestingly, hippocampal 5-HT levels were significantly reduced, which may not be directly related to improved memory but rather to the central regulation of stress-related and exploratory behaviors. Although several studies have demonstrated that prebiotics can enhance cognition in piglets, their effects on exploratory or motor behavior appear limited in preterm pigs [65,133]. For instance, supplementation with short-chain galacto-oligosaccharides and long-chain fructo-oligosaccharides, along with *Bifidobacterium breve*, improved cognitive performance but had minimal impact on locomotion or exploration. This might be due to the synbiotic-induced acceleration of brain maturation and dendritic arborization.

Although the above results all suggest that prebiotics from multiple sources can improve cognitive ability, a reasonable inference has not yet been made regarding the way prebiotics improving cognition, and more studies are needed to prove whether prebiotics regulate abnormal behaviors.

### 6.4. Nutrient Adjustments

Apart from direct modulation of the gut microbiota, it can also be indirectly modulated through nutrient adjustments. These nutrients include proteins, lipids, carbohydrates, minerals, dietary fiber, vitamins, and water. Proteins, minerals, dietary fiber, and vitamins can influence the gut microbiota, thereby positively affecting the physiological and psychological state of the animal and thus enhancing animal welfare.

#### 6.4.1. Amino Acid

Amino acids have been shown to influence behavior through modulation of the gut microbiota. L-tyrosine, by reshaping the gut microbiota, regulates gut homeostasis, prevents hippocampal degeneration, and alleviates autism-like behaviors in mice [134]. L-proline enhances the abundance of *Akkermansia*, while concurrently reducing the abundance of *Lachnospiraceae_UCG-006*, *Candidatus_Saccharimonas*, and *Ileibacterium* [135]. This results in a decrease in pathways related to pyruvate metabolism, taurine and secondary taurine metabolism, and nucleotide metabolism, while pathways involved in the biosynthesis of phenylalanine, tyrosine, and tryptophan and α-linolenic acid metabolism increase. These changes reverse pathological alterations, oxidative stress, and BBB damage in the PFC, and reduce systemic inflammation in a valproic acid-induced ASD mouse model, thereby improving autism-like behaviors [136].

Amino acids are also precursors of key neurotransmitters in the MGBA. For example, tryptophan is the precursor of 5-HT, a major neurotransmitter implicated in MGBA-mediated behavioral regulation, and has been hypothesized to play a role in TB [73]. Supplementation in the diet can effectively increase 5-HT in the hypothalamus. Large neutral amino acids (LNAAs), however, compete with tryptophan to pass through the BBB. Reducing the intake of LNAA can effectively increase the content of tryptophan in saliva. Nevertheless, when the reduction amount is too high, it will increase fighting behaviors. This might be because excessive 5-HT can enhance behavioral activities [137]. Some studies have hypothesized that reducing protein/amino acid levels may affect TB through the immune pathway in the MGBA. Moreover, protein deficiency can lead to depression or aggression, while excessive protein can increase depression [73]. Therefore, the impact of protein/amino acids on behavior needs more in-depth exploration.

#### 6.4.2. Mineral

During the growth and reproductive stages of pigs, trace minerals such as iron, iodine, magnesium, and zinc play essential roles not only in physiological development but also in the regulation of behavior. When iron is lacking in the diet, it affects the iron content in the blood, liver, and hippocampus, further reducing the memory abilities and spatial learning of piglets. Although in this study it has not been correlated with gut microbiota, increasing iron intake can increase the relative abundances of *Lactobacillus* and *Lactobacillus amylovorus* [138]. Indeed, *Lactobacillus* can improve memory. In another study, pigs were subjected to two types of mineral-deficient diets: one lacking all mineral supplements and the other deficient only in iodized salt. Pigs fed the iodine-deficient diet showed a heightened interest in bleeding tails, suggesting that iodine craving may increase attraction to injured pen mates and potentially promote TB [139]. However, research on the behavioral effects of individual minerals in pigs remains limited, and conclusions often rely on extrapolations from rodent studies. Research has already found that magnesium ions are essential minerals for neuronal health. They can regulate ion channels, maintain the structural integrity of cell membranes, and synaptic plasticity. The deficiency of magnesium ions can lead to anxiety-like behaviors and alterations of gut microbiota in mice, which is associated with the reduction in interleukin-6 in the hippocampus [140]. Some studies have found that zinc deficiency in rodents can affect memory, lead to depression, and increase aggression, and that zinc can influence the gut–brain axis [141]. There are also studies revealing that zinc oxide nanoparticles can cause neurobehavioral disorders through the MGBA, which may be related to excessive 5-HT to some extent. However, the impact of zinc on pig behaviors has not yet been determined [142].

#### 6.4.3. Fiber

Different types of functional dietary fibers exert varying effects on pig behavior. Some fibers regulate behavior by increasing satiety through their water-holding and swelling capacity. Research has examined the supplementation of grass silage except straw [143]. Although the occurrence of uninjured ears and tails in both treatments were insufficient for statistical evaluation, the group with access to silage demonstrated virtually no tail damage and enhanced exploratory activities. Supplementation with 5% resistant starch, known for its strong swelling capacity in gestational diets increased postprandial satiety, alleviated stress responses, and reduced abnormal behavior in sows [144]. In another study, different fiber sources were compared in gestating sow diets (resistant starch 11%, beet pulp 27%, and soybean hulls 19%) [145]. Sows fed soybean hulls spent the most time resting, while beet pulp feeding increased standing behavior. Both resistant starch and soybean hulls improved welfare by enhancing satiety and reducing aggression in restricted-fed pregnant sows.

In addition to mechanical effects, dietary fiber serves as a substrate for microbial fermentation, thus influencing both gut microbiota composition and microbial metabolites, with subsequent impacts on animal welfare. In mice, long-term fiber deprivation leads to cognitive deficits, which have been linked to altered gut microbiota profiles. This cognitive impairment is associated with reduced SCFA production, activation of hippocampal microglia, increased synaptic phagocytosis, and neuroinflammation [146]. It has been proposed that the relationship between the kind and quantity of dietary fiber and TB is linked to the MGBA regulation of gut health and stress responsiveness. This is because dietary fiber can modify gut microbiota, and a connection exists between gut microbiota and TB behavior [73]. However, direct evidence linking microbial metabolites derived from fiber fermentation to behavioral outcomes in pigs remains scarce. Notably, many fiber types associated with behavioral modulation are prebiotics. For instance, dietary supplementation with oligofructose has been shown to improve recognition memory in pigs during delayed testing, which was linked to changes in hippocampal gene expression involving dopaminergic, GABAergic, cholinergic pathways, as well as genes related to cell adhesion and chromatin remodeling [131].

#### 6.4.4. Vitamin

A growing body of research has demonstrated that vitamins are closely associated with anxiety and depression-like behaviors, as well as ASD, primarily through modulation of the MGBA. Vitamin B6 has been shown to alleviate anxiety and depression-like behaviors induced by chronic restraint stress in rats, likely by correcting stress-associated disruptions in gut microbiota and vitamin B6 metabolism, independent of cortisol levels [147]. Vitamin D is an important vitamin with multiple applications [148,149]. It is a key modulator of behavior, exerting its effects by altering gut microbial composition and regulating 5-HT synthesis. Numerous studies have reported that vitamin D supplementation can mitigate anxiety and depressive symptoms [150]. Developmental vitamin D deficiency has also been identified as a contributing factor in the pathogenesis of ASD. This link may be associated with alterations in gut microbiota and increased levels of propionic acid in the ileum [151]. Early-life deficiencies in several vitamins, including vitamins A, B12, D, and K, have been associated with increased risk of neurodevelopmental disorders such as ASD. These effects are believed to involve immune regulatory mechanisms within the MGBA [152]. Further supporting this, FMT from human donors into ASD mouse models has been shown to improve social behavior, with vitamin B6 metabolism implicated as a key mediator of this effect [153]. Direct supplementation with vitamin B6 in ASD mice further confirmed its beneficial effects on social behavior Although most of these studies have been conducted in rodent models, similar findings have been reported in humans, where deficiencies in vitamins B1 and B2 have been associated with anxiety, stress, and poor sleep quality, possibly via the MGBA [154]. Given the physiological similarities between pigs and humans, it is plausible that vitamin supplementation may also influence pig behavior through microbial modulation, thereby offering a promising strategy for improving animal welfare in pig production systems.

## 7. Conclusions

In intensive farming systems, abnormal behavior in pigs is a significant indicator of compromised welfare. The discovery of the MGBA provides a novel perspective for understanding behavior regulation. This review highlights that gut microbiota communicates bidirectionally with the pig’s brain through immune, endocrine, and neural pathways, directly or indirectly influencing behavioral outcomes. It suggests that strategies targeting the optimization of gut microbiota, such as the use of probiotics, could potentially improve pig welfare. Although significant progress has been made in understanding the mechanisms by which the MGBA regulates animal behavior, notable limitations remain in current research. First, much of the mechanistic evidence comes from rodent models, and significant differences in microbial composition and behavioral responses have been observed between pigs and rodents when subjected to the same interventions, suggesting that direct extrapolation of findings from rodent models to pigs may lead to biased conclusions [155,156]. Second, existing studies mainly rely on static association analyses, lacking continuous tracking of the dynamic interactions between the microbiota, metabolites, and behavior, thus limiting the ability to determine causal relationships.

To address these gaps, future research should integrate multi-omics approaches with behavioral monitoring technologies to develop a dynamic research framework. By screening for neuroactive metabolites through metabolomics, coupled with automated behavioral monitoring to quantify phenotypes such as feeding and aggression in pigs, the key mechanisms can be further validated. This approach will enable a shift from correlation to causation, providing theoretical support for developing targeted microbiota interventions, ultimately offering effective strategies to improve the welfare of pigs in intensive farming systems and promoting the sustainable development of the pig farming industry.

## Figures and Tables

**Table 2:** Influence of gut microbiota on pig behavior.

Behavior	Microbiota	Influence	Reference
Feeding Behavior	*Marvinbryantia*, *Dorea*, *Blautia*, and *Ruminococcaceae_UCG-014*	Promote pig feeding.	[59]
	*Christensenellaceae_R-7_group*, *Family_XIII_AD3011_group*, and *Ruminococcaceae_UCG-004*	Inhibition of pig feeding.	
Excretion Behavior	*Prevotella*, *Sutterella*, and *Campylobacter*, and *Clostridiaceae*	Diarrhea in newborn piglets.	[61]
Group Living Behavior	*Faecalibacterium*, *Peptococcus*, and *Oliverpabstia*	Dominant pigs of cohort.	[62]
	*Holdemanella* and *Acetitomaculum*	Subordinate pigs of cohort.	
Sexual Behavior	*Anaerovibrio*, *Succinivibrio*, *Treponema*, *Oribacterium*, *Faecalibacterium*, and *Prevotella*	Successful puberty in reserve sows.	[63]
	*Bacteroidia*, *Lactobacillaceae*, and *Lactobacillus*	Restoration of normal estrus in sows.	[64]
Exploratory Behavior	*Coprococcus 3*, *Eubacterium*, and *Coprostanoligenes*	Piglets squeal in open field test.	[60]
	*Atopobium* and *UBA1819*	Piglets displayed nosing behaviour and total exploration behaviour.	
Abnormal Behavior	*Lachnospiraceae*, *Ruminococcaceae* and *Clostridiales Family XIII*	Piglets show tail biting behavior.	[55]
	*Lactobacillus*	Reducing the chances of pigs biting and being bitten on the tail.	[56]
After-effect Behavior	*Clostridium XIVa* and *XVIII*, *Faecalicoccus* and *Ruminococcaceae*	Reducing the time pig took to successfully cross the fence barrier.	[65]

## Data Availability

No data were used for the research described in the article.

## Abbreviations

The following abbreviations are used in this manuscript:

5-HT	5-hydroxytryptamine
AHR	aryl hydrocarbon receptor
ASD	autism spectrum disorder
BBB	blood–brain barrier
CNS	central nervous system
ENS	enteric nervous system
FMT	fecal microbiota transplantation
GABA	γ-aminobutyric acid
GF	germ-free
HFD	high-fat diet
HPA	hypothalamic–pituitary–adrenal
LNAA	large neutral amino acids
LPS	lipopolysaccharides
MGBA	microbiota–gut–brain axis
PFC	prefrontal cortex
SCFA	short-chain fatty acid
TB	tail biting
